# Auxin driven indoleamine biosynthesis and the role of tryptophan as an inductive signal in *Hypericum perforatum* (L.)

**DOI:** 10.1371/journal.pone.0223878

**Published:** 2019-10-17

**Authors:** Lauren A. E. Erland, Praveen Saxena

**Affiliations:** Department of Plant Agriculture, Gosling Research Institute for Plant Preservation, University of Guelph, Guelph, Ontario, Canada; United Arab Emirates University, UNITED ARAB EMIRATES

## Abstract

In the 60 years since Skoog and Miller first reported the chemical redirection of plant growth the underlying biochemical mechanisms are still poorly understood, with one challenge being the capacity for applied growth regulators to act indirectly or be metabolized to active phytohormones. We hypothesized that tryptophan is metabolized to auxin, melatonin or serotonin inducing organogenesis in St. John’s wort (*Hypericum perforatum* L.). Root explants from two germplasm lines of St. John’s wort with altered melatonin metabolism and wildtype were incubated with auxin or tryptophan for 24, 48 or 72 h to induce regeneration. In wildtype, tryptophan had little effect on the indoleamine pathway, and was found to promote primary growth, suggesting excess tryptophan moved quickly through various secondary metabolite pathways and protein synthesis. In lines 4 and 112 tryptophan was associated with modified morphogenesis, indoleamine and auxin levels. Incubation with tryptophan increased shoot organogenesis while incubation with auxin led to root regeneration. The established paradigm of thought views tryptophan primarily as a precursor for auxin and indoleamines, among other metabolites, and mediation of auxin action by the indoleamines as a one-way interaction. We propose that these processes run in both directions with auxin modifying indoleamine biosynthesis and the melatonin:serotonin balance contributing to its effects on plant morphogenesis, and that tryptophan also functions as an inductive signal to mediate diverse phytochemical and morphogenetic pathways.

## Introduction

Tryptophan is an aromatic amino acid, produced from chorismate via the shikimate pathway [1]. It is incorporated into proteins during synthesis and it provides the structural backbone for hundreds of thousands of plant secondary metabolites including the indoleamines [2], auxin (indole-3-acetic acid; IAA)[3,4], alkaloids [5], benzoxazinoids [6], and glucosinolates [7]. The majority of studies on tryptophan in plants have examined tryptophan supplementation as a means of up-regulating production of commercially or biologically valuable downstream metabolites, or have focused on the capacity for tryptophan to enhance growth through increased protein synthesis [8,9]. Some studies have, however, noted that tryptophan supplementation can affect growth characteristics and morphogenesis in plants. For instance, tryptophan (20–240 μM), as well as another amino acid, proline, have been found to enhance somatic embryogenesis in rice (*Oryza sativa* L. cv. Pusa 196)[10]. Tryptophan (100–200 μM) has also been found to be effective in promoting callus formation and plantlet production from callus in several cultivars of Malaysian upland rice (cv.’s Kusan, Lamsan, Siam). In *Catharanthus roseus* (L.) application of tryptophan has also been found to improve growth and photosynthetic capacity, and these results were suggested to be partially due to modulation of cytokinin levels, as well as increasing auxin (IAA), gibberellic acid and abscisic acid levels [11]. Tryptophan levels have been found to be highest in meristematic tissues as well as in storage tissues such as seeds and tubers [12]. Some early studies on tryptophan found that levels were greatest in developing reproductive structures, seeds and seedlings [12–15] though these effects have largely been attributed to a concurrent increase in auxin levels.

Interestingly, the observed responses for tryptophan are similar to those reported for another important group of growth regulating tryptophan metabolites: melatonin and serotonin [16–20]. Melatonin (N-acetyl-5-methoxytryptamine) and serotonin (5-hydroxytrytamine) are indoleamines first discovered in the mammalian system, but which have now been found to be produced by almost every form of life, including across the plant kingdom [21]. Melatonin and serotonin play important roles at every stage in the plant life cycle including germination, seedling development, vegetative growth, floral patterning and reproductive growth, as well as, mitigating diverse biotic and abiotic stresses [21–24]. They are well established as potent antioxidants, which may provide beneficial physiological responses to environmental stresses, as they act both as direct antioxidants and upregulate other antioxidant pathways [25,26].

Recently the indoleamines have been shown to play important roles in plant morphogenesis. Crosstalk between melatonin, serotonin and other plant growth regulator pathways such as cytokinins and auxin, has been demonstrated in *Hypericum perforatum* (L.), commonly known as St. John’s wort (SJW) [27,28], and several other plant species [29–33]. Additionally, it has been proposed that melatonin and serotonin act in concert with each other, and their precursors, to mediate their effects, with melatonin having auxin-like effects and serotonin cytokinin-like effects [21]. Serotonin has been reported to inhibit auxin activity at both primary and adventitious root meristems, and in lateral root primordia of *Arabidopsis thaliana* [32]. As inhibition of auxin function is a well-documented mechanism of cytokinin function, this supports the analogy of serotonin functioning in the place of the cytokinin in the melatonin:serotonin balance. Serotonin has also been found to interact with jasmonic acid and ethylene in *Arabidopsis* to modify root architecture [34]. Our previous study in SJW found that indoleamine-induced *de novo* shoot organogenesis was linked with increased zeatin levels [27]. Additionally, recent literature has found in x-ray crystallography studies that melatonin interacts with zeatin, a natural cytokinin, at the binding site of the PR-10 protein cloned from yellow lupine (*Lupinus luteus* L.) [35] [36], further suggesting a significant competitive interaction between melatonin and cytokinins. Abscisic acid, salicylic acid and gibberellic acid have all also been found to interact with indoleamines, though the majority of the available information on these interactions is in response to abiotic stress tolerance [27,37,38].

**Fig 1:**
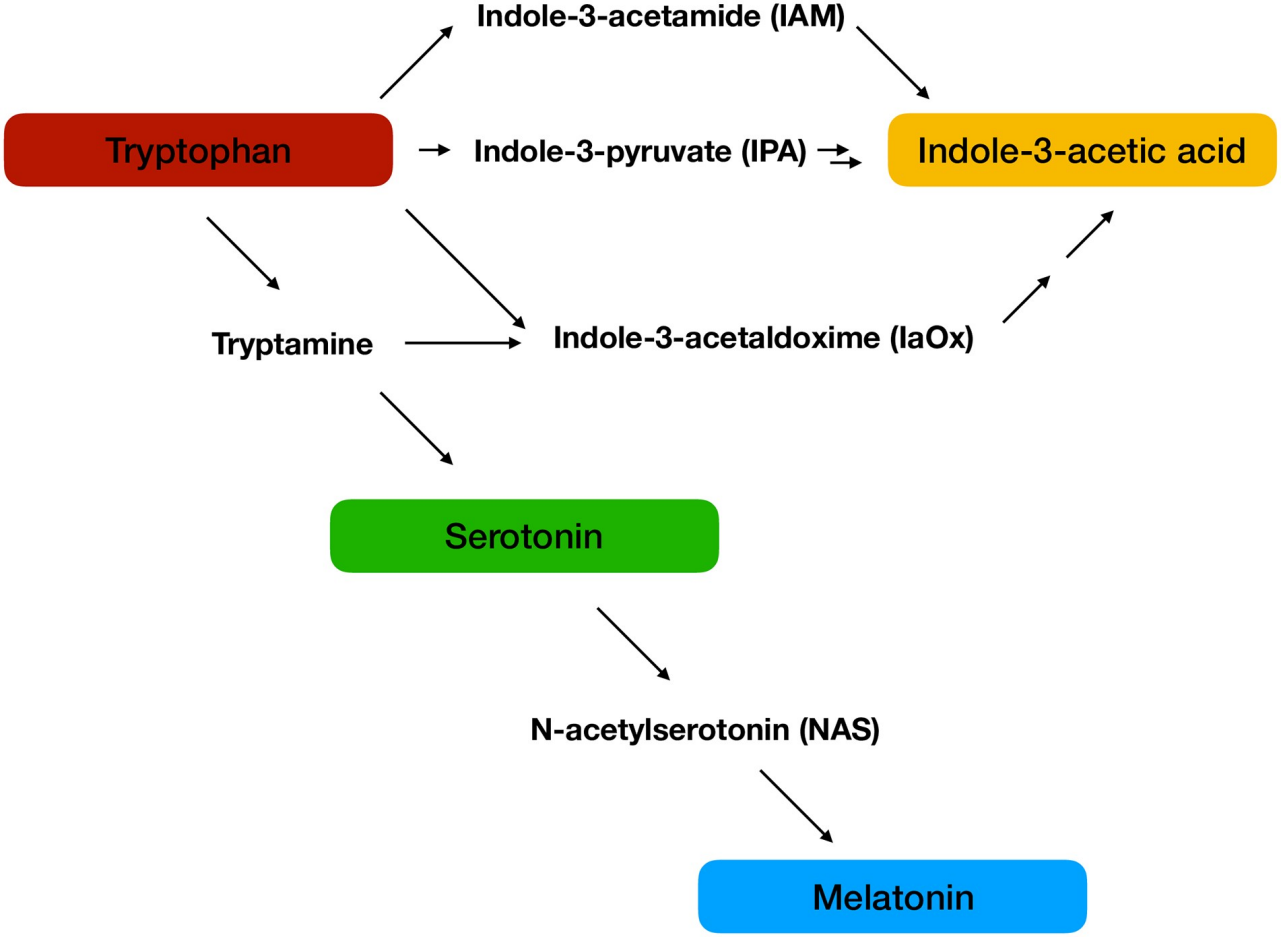
Summary of tryptophan metabolism to indoleamines and auxin.

In plants the indoleamines are synthesized from tryptophan (Fig 1); in the main biosynthetic pathway tryptophan is first converted to tryptamine by tryptophan decarboxylase (TDC) [39]. It is interesting to note that though TDC is recognized as tryptophan decarboxylase, it is actually an aromatic amino acid decarboxylase in plants, which is capable of decarboxylating other aromatic amino acids, such as tyramine, making this a complex and highly regulated step [40,41]. In the indoleamine pathway after conversion of tryptophan to tryptamine, tryptamine-5-hydroxylase (T-5-H) then converts tryptamine to serotonin [42]. Serotonin is then acetylated by serotonin-N-acetyltransferase (SNAT)[43] to form N-acetylserotonin (NAS) which is finally converted to melatonin by acetylserotonin-O-methyltransferase (ASMT) [44]. There are an increasing number of alternative biosynthetic routes, however, which have been elucidated [45–47] as well as several enzymes in the pathway which have been found to be capable of the reverse reaction [48], making this a highly adaptive and dynamic pathway. In a recent study [27] it was found that tryptophan was capable of mediating plant growth responses in both root and shoot explants of SJW. This response may be due to diverse phenomena, as tryptophan serves not only as a building block in protein synthesis, but is also a common precursor for melatonin and serotonin, as well as the classical phytohormone auxin [49].

**Fig 2:**
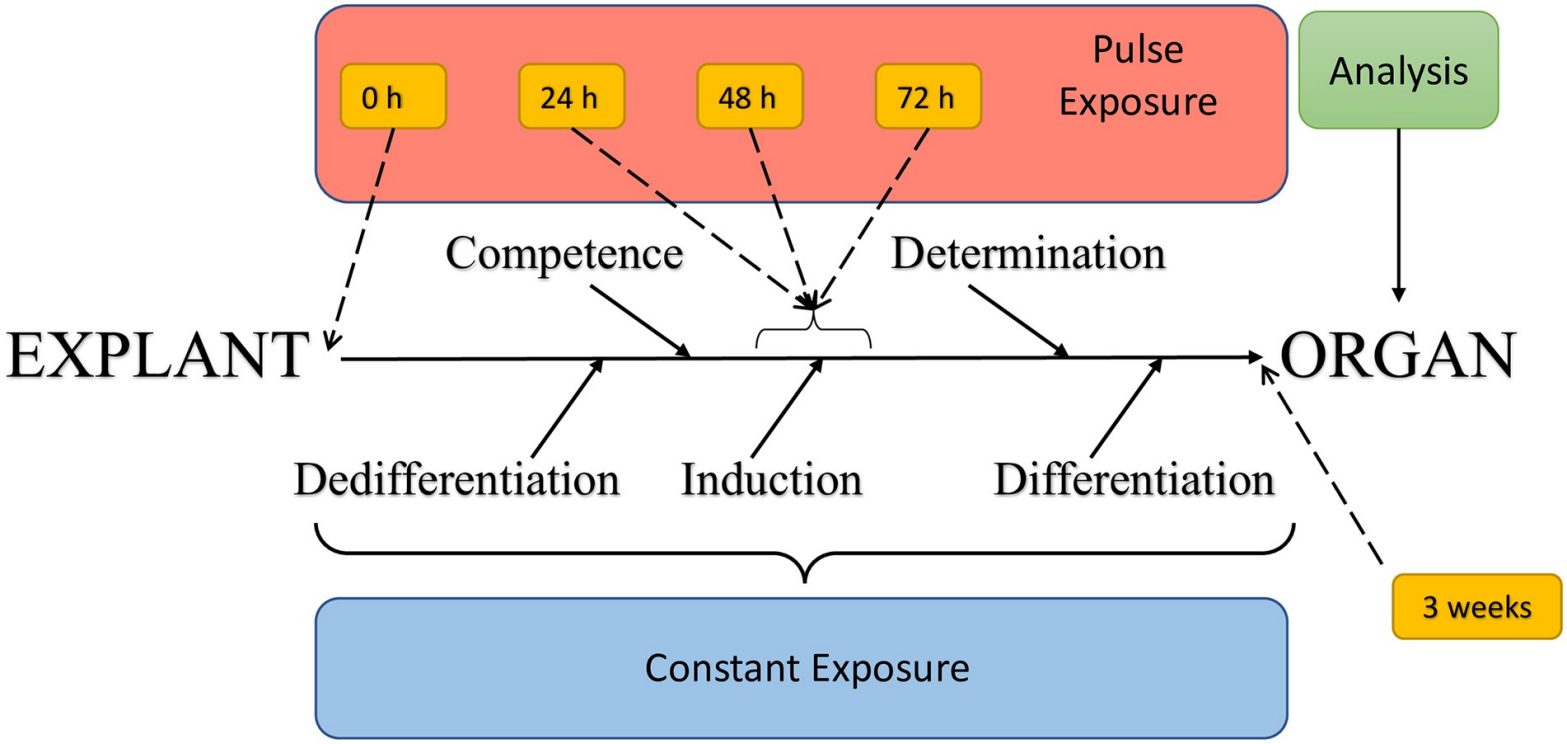
Overview of experimental timing as it relates to the plant morphogenetic process from explant to organ production.

Plant morphogenesis is a complex physiological process that is not completely understood. An explant is cleaved from the maternal plant tissue to disrupt the flow of regulatory signals between cells [50] and an inductive signal is applied. Cells then go through a process of dedifferentiation to a less committed, more flexible or plastic developmental state and are competent to receive an inductive signal that determines the route of regeneration resulting in either a root or a shoot [50] (Fig 2). To date, the exact role of tryptophan, melatonin or serotonin in this process has not been elucidated, and it is not clear if these metabolites are the inductive signals or part of the morphogenesis expression machinery.

We hypothesized that increased supply of tryptophan will increase the carbon flow to the auxin/melatonin biosynthetic pathways, thereby inducing morphogenesis in three lines of SJW: wild-type (WT) and lines 4 and 112. To investigate this hypothesis, we flooded the amino acid pool of tryptophan with a bolus dose during the induction phase of regeneration and measured the long-term morphogenetic responses of the plant tissues. Our data show that incubation with tryptophan, even at 24 h exposure, increased *de novo* shoot organogenesis, while incubation with auxin led to *de novo* root regeneration. We propose that auxin modifies indoleamine biosynthesis and the balance between melatonin and serotonin, which may contribute to its effects on plant morphogenesis. Additionally, we propose that tryptophan functions as an inductive signal which can be directed to mediate diverse phytochemical pathways, including but not limited to, the indoleamines and auxin.

## Materials and methods

### Germplasm lines

St. John’s wort (*Hypericum perforatum* L.) cultures were acquired from the germplasm bank maintained at the Gosling Research Institute for Plant Preservation (GRIPP), University of Guelph, Guelph, Ontario. The germplasm lines were generated as described previously [51,52]. Briefly, germplasm line 4 was the result of EMU mutagenesis, while line 112 was the result of anther culture. These lines were compared with WT, which originated from *H*. *perforatum* cv ‘Anthos’ (Richter’s. Herbs, Ontario, Canada), for these experiments [27,51,52]. Germplasm lines have been maintained in perpetual axenic culture on a media consisting of Murashige and Skoog [53] basal salts with 1 mL L^-1^ Gamborg’s B5 vitamins, 3% (w/v) sucrose, with pH adjusted to 5.7, solidified with 0.22% (w/v) phytagel and autoclaved at 121 °C at 18 psi for 20 min (hereafter referred to as MSO) in GA7 culture vessels (Caisson Labs, USA). All cultures are maintained in a controlled environment culture room at 25 ± 2 °C under a 16-h photoperiod provided by cool white fluorescent lamps (Osram Sylvania Ltd., Mississauga, Ontario, Canada) at light intensity of 40 μmol·m^−2^·s^−1^.

### Constant exposure experiments

Root explants (7–8 mm segments from primary roots, not within 0.5 cm of root tip or base) from all three lines were cultured onto test media in Petri plates (eight explants per plate; 60 x 15 mm Polystyrene disposable sterile Petri plates). Test media were prepared as described for MSO with the addition of 10 μM tryptophan, or IAA. Tryptophan stock solutions were prepared by dissolving in 1 M HCl and diluting with distilled water. IAA stock solutions were prepared by dissolving 1 M NaOH diluting in distilled water. Stock solutions were filter sterilized and added to medium after autoclaving, and once the medium had reached a temperature of less than 60 °C. MSO controls were included with each experiment. Cultures were incubated at 26 °C under cool white fluorescent lights (as described above) with a 16-h photoperiod for two weeks. After 21 days, growth data including: number of internodes, number and height of shoots, number and length of roots, and presence of microshoots (defined as shoots under 1 mm in height) was collected and collated for statistical analyses.

### Liquid pulse exposure

Liquid MSO medium was prepared as described above without the gelling agent and allocated across sterilized 50 mL Erlenmeyer flasks. Root explants were excised from stock plants of WT and germplasm lines 4 and 112 and incubated in 20 mL liquid MSO supplemented with 0 μM or 10 μM tryptophan or auxin for 0, 24, 48 or 72 h in flasks (8 per flask) with gentle shaking on a shaker table (80 rpm). After the incubation period, explants were subcultured onto solid MSO medium in Petri dishes (30 x 15 mm polystyrene disposable sterile Petri plates, VWR, Mississauga, Canada) and incubated in the controlled environment chamber at 26 °C under cool white fluorescent lights (~40 μm m^-2^ s^-1^, Osram Sylvania Ltd., Mississauga, Ontario, Canada) with a 16 h photoperiod for 21 days. After 21 days, fresh weight and the rate of differentiation of cells into new organs was visually observed and data collated for statistical analyses. Regenerating explants were harvested, rinsed with sterile distilled water and patted dry. Explants were pooled by plate in pre-weighed Eppendorf tubes and stored at– 80°C for chemical analysis.

### Determination of endogenous auxin and indoleamine content

Samples were analyzed for indoleamine and plant growth regulator content as described by Erland *et al*. (2016, 2018) with slight modification [54,55]. In brief, samples were first ground in liquid nitrogen to a fine powder, then suspended in 0.25 mL of extraction solvent comprised of 50% methanol (MS Grade, Fisher Scientific, Canada) and 4% acetic acid (glacial, Fisher Scientific, Canada) in Milli-Q water. Samples were then sonicated (3510R-DTH, Branson, USA) for 30 min on ice and spun down (2 min, 13000 rpm) in order to remove the supernatant. Supernatant was then filtered through a 0.22 μm centrifuge filter (Millipore; 1 min, 13 0000 rpm) with the flow through diluted five-fold in Milli-Q water.

Indoleamines were separated by reverse phase liquid chromatography (Acquity Classic ultra-performance liquid chromatography system; Waters Canada, Mississauga, ON) by injection of a 5 μL aliquot of sample onto an Acquity BEH Column (2.1 x 50 mm, i.d. 2.1mm, 1.7 μm; Waters). For quantification of melatonin, tryptophan and IAA, analytes were separated with a gradient of solvents A (10 mM ammonium acetate pH 9, adjusted with ammonium hydroxide) and B (100% methanol) with initial conditions at 95% A (5% B) increased to 5% A (95% B) over 4.5 min using an Empower curve of 8. The column temperature was 40 °C and flow rate was 0.5 mL/min. For quantification of serotonin, samples were run with a gradient of solvents A (0.1% formic acid; Waters, Mississauga, Canada) and B (100% methanol) with initial conditions 95% A (5% B) increased to 5% A (95% B) over 1.5 min using an Empower curve of 6. The column temperature was 30 °C and flow rate was 0.5 mL/min. Metabolite peaks were identified by comparison to authentic standards and quantified by comparison to a standard curve as described previously [54,55].

Phytochemicals were detected with single quadrupole mass spectrometer (Waters, QDa performance model) using single ion recording mode in positive mode. Serotonin and IAA were quantified at a m/z of 177, melatonin at 233, and tryptophan at 205. In all cases probe temperature was set to 500°C with a gain of 5; capillary voltage was set to 0.5 kV. The limit of detection was 6.10 ng/mL, 95.3 pg/mL, 97.6 ng/mL and 0.61 ng/mL for tryptophan, melatonin, serotonin and auxin, respectively, while the limits of quantification were 6.10 ng/mL, 0.38 ng/mL, 0.39 μg/mL and 6.1 ng/mL, respectively [54,55].

### Experimental design and statistical analysis

Two parallel sets of experiments were designed to test the plant morphogenic responses from induction to expression and the biochemical responses to tryptophan and auxin application (Fig 2).

All treatments contained 8 pseudoreplicates (root sections) per plate, with 4 replicate Petri plates (flasks) per treatment, per germplasm line. To account for variability of explants across each plate, results from pseudoreplicates were averaged across a plate and plate means were used as replicates in further statistical analyses. Experiments were duplicated twice and results were combined. All statistical analyses and plotting were performed in Prism (v7.0; GraphPad, Inc).

Linear regression analysis was performed on growth data within treatment groups for each time point collected (24, 48 and 72 h) and the Shapiro Wilkes Data was not found to be universally normal and was therefore analyzed using a two-tailed Mann-Whitney non-parametric t-test to compare between MSO controls and tryptophan or IAA treated controls for each time point collected (Constant, 24, 48 or 72 h) with α = 0.05.

For growth regulator analysis, in instances where the compound was below the limit of detection in some replicates within a treatment group but was detected in others the mean of zero and the limit of detection was used in lieu of zero. In treatment groups where the compound was not detected in any of the replicates zero was used in all replicates for this treatment group. Data was not found to be universally normal and was analyzed via a two-tailed Mann-Whitney non-parametric t-test as described for growth data.

## Results

### Effects of tryptophan treatment on indoleamine metabolism

**Fig 3:**
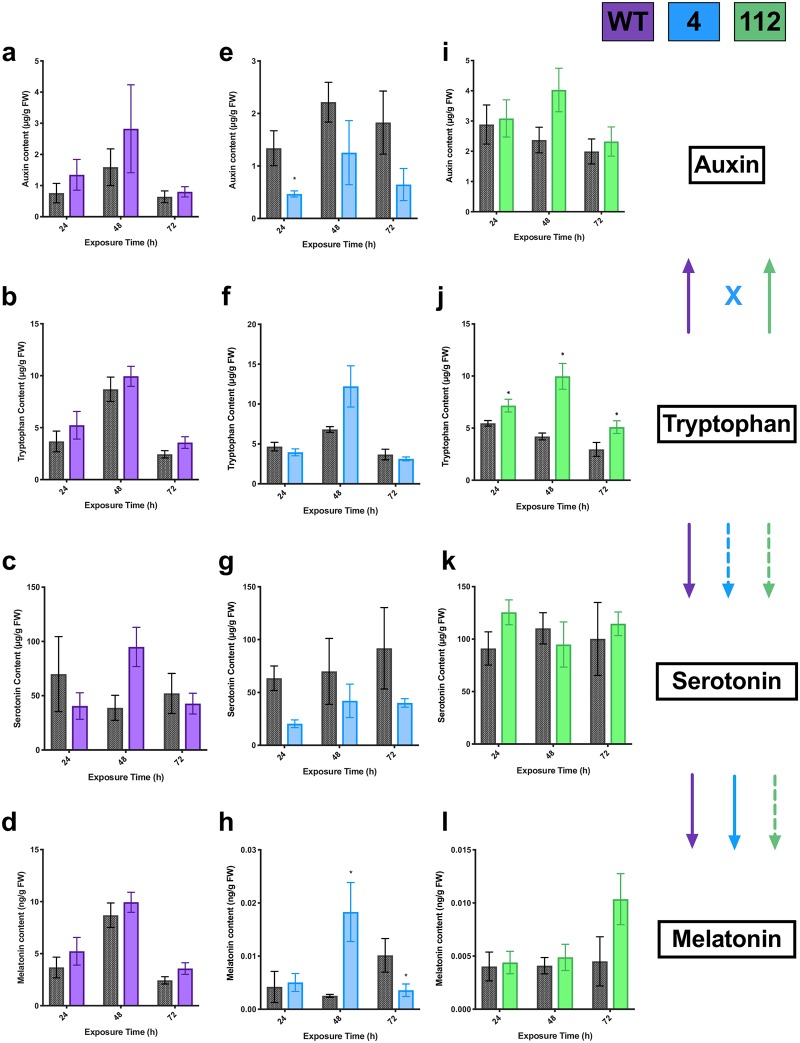
Effects of tryptophan treatment (10 μM) on endogenous tryptophan (b, f, j), auxin (indole-3-acetic acid; a, e, i), serotonin (c, g, k), and melatonin (d, h, l) levels in three lines of St. John’s wort roots wildtype (WT; purple), 4 (blue) and 112 (green). * indicates treatment is significantly different than control (MSO; grey) at a given exposure time by t-test, with α = 0.05 (n = 5). Solid lines indicate tryptophan conversion to the metabolite, hashed lines are a putative conversion pathway, and ‘x’ indicates that the conversion is interrupted.

WT SJW cultures fed tryptophan did not have a significant increase in the tryptophan metabolic pool after three weeks of growth (Fig 3b) and tryptophan feeding had no significant long-term effects on serotonin, melatonin or auxin content (Fig 3), though there was a trend towards an increase in serotonin content in cultures exposed for 48h (p = 0.0571, S1 Table). It is possible that this is due to rapid conversion of tryptophan to other secondary metabolites or protein synthesis, or feedback inhibition. In contrast, tryptophan treatment significantly increased the overall tryptophan pool size in cultures of line 112 (Fig 3j) as well as a slight but insignificant increase in auxin content after 48 h of exposure (p = 0.0571; Fig 3i, S1 Table), with no change in serotonin (Fig 3k) or melatonin (Fig 3l) levels observed. It is possible that a downstream disruption in the indoleamine pathway may contribute to the maintenance of higher tryptophan content even after three weeks in these cultures. The melatonin content in lines 4 and 112 was 1000 times lower than in both control and treated WT cultures (Fig 3d, 3h and 3l), suggesting that the indoleamine biosynthetic pathway has been disrupted in these lines as previously described [27]. Similar to line 112, cultures of line 4 showed an increase in the tryptophan pool size after 48 h of exposure (Fig 3f), but inversely showed a significant decrease in auxin levels, though this decrease was only significant at 24 h (Fig 3e; p_24h_ = 0.029; p_48h_ = 0.114; S1 Table), suggesting a potential feedback mechanism. Melatonin content showed a significant increase after 48 h in line 4, however, it decreased significantly at 72 h (Fig 3h); while serotonin did not show a significant decrease in any treatment. These results suggest that line 4 may have a differential flow of carbon through the pathway. This is particularly noticeable when compared to line 112 where increased bolus doses of tryptophan does not have an effect on downstream melatonin or serotonin levels indicating a clear disruption in the indoleamine biosynthetic pathway.

### Effects of tryptophan treatment on growth and morphogenesis

**Fig 4:**
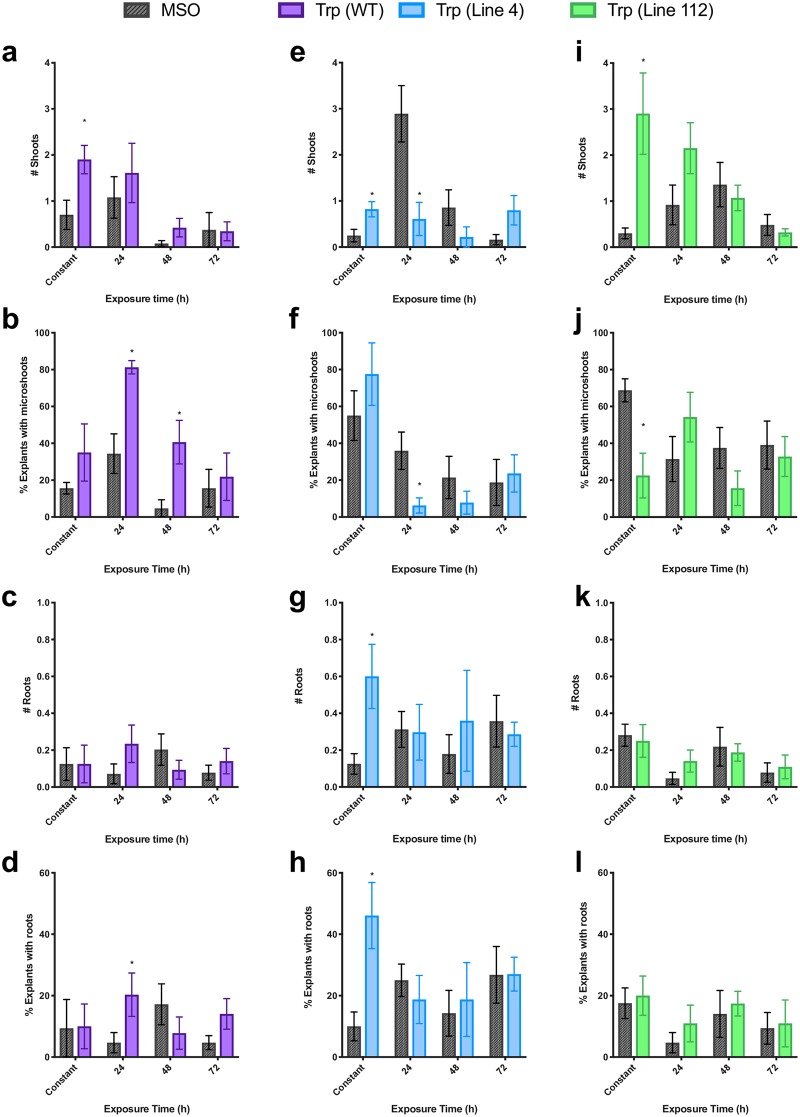
Effects of tryptophan treatment (10 μM) on morphogenesis as measured by mean number of shoots produced per explant (a, e, i), mean percent of explants with the presence of microshoots, defined as shoots under 1 mm in length (b, f, j), mean number of roots produced per explant (c, g, k) and mean percent of explants producing roots (d, h, l) in three lines of St. John’s wort roots: Wildtype (purple; a-d), 4 (blue; e-h) and 112 (green; i-l). * indicates treatment is significantly different than control (MSO; grey) at a given exposure time by t-test, with α = 0.05 (n = 5).

**Fig 5:**
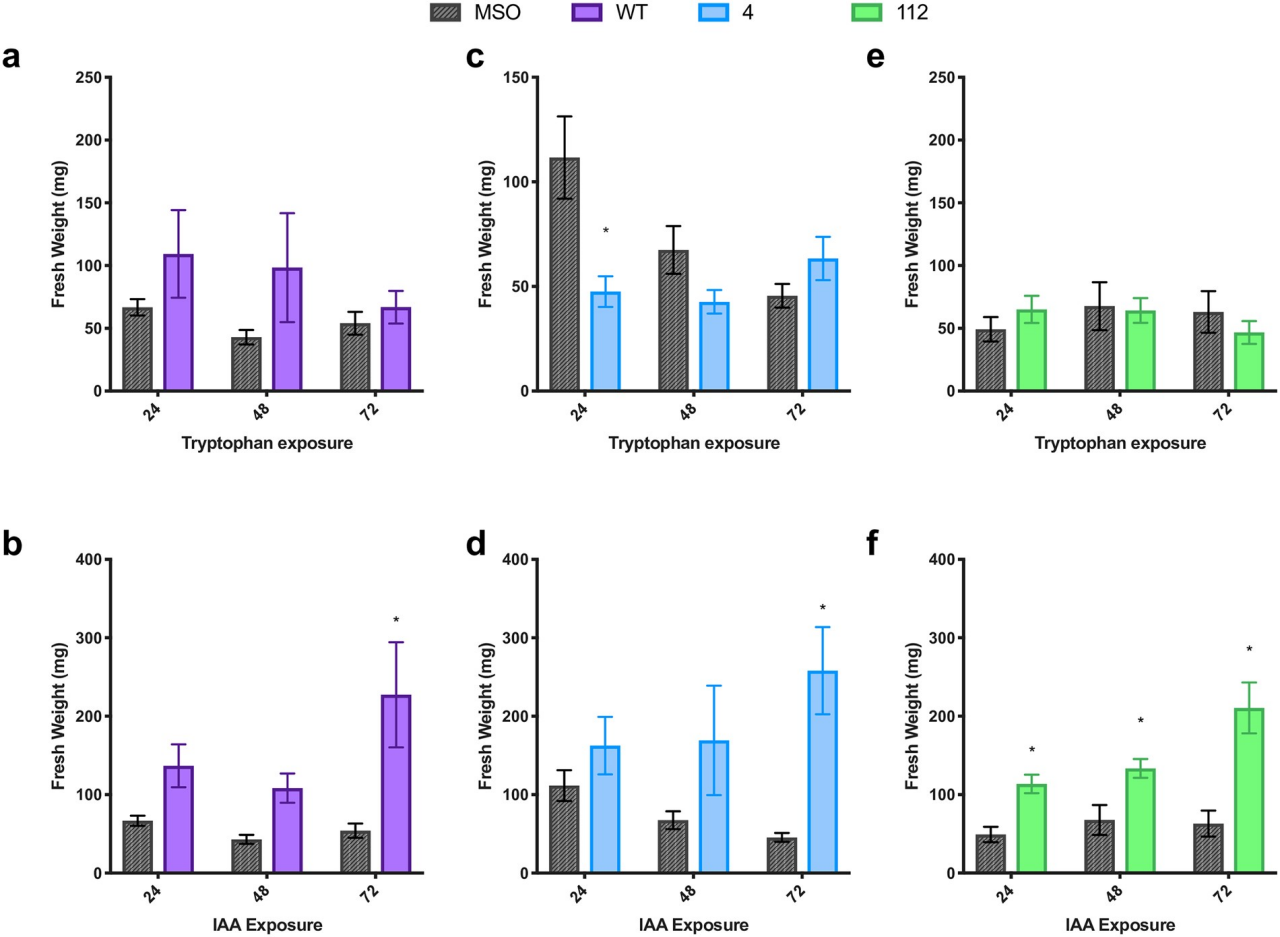
Effects of tryptophan (a, c, e) and auxin (b, d, f) treatment on fresh weight in three lines of St. John’s wort (WT; purple); 4 (blue) and 112 (green). * indicates treatment is significantly different than control (MSO;grey) at a given exposure time by t-test, with α = 0.05 (n = 5).

In the liquid pulse exposure experiments, tryptophan exposure increased *de novo* shoot organogenesis in line 112 (Fig 4i and 4j) and WT (Fig 4a and 4b), particularly with shorter periods of exposure to the bolus amino acid (e.g. 24 and 48 h). Longer incubations of tissues in tryptophan solutions resulted in a decline in regeneration. Tryptophan significantly increased shoot production from root explants of 112 after 24 h of exposure (Fig 4i), while a more modest increase was seen in microshoot (shoots < 1 mm in length) production (Fig 4j; S2 Table; p_24h_ = 0.0128; p_48h_ = 0.067). In WT, total number of shoots was not significantly increased (Fig 4a; p = 0.106), although the percentage of explants producing microshoots was significantly higher at 24 h and 48 h exposure times (Fig 4b), with a linear decline in % microshoots with increasing exposure time (Table 1). In contrast, tryptophan appeared to have an inhibitory effect on shoot regeneration in line 4 (Fig 4e and 4f), with the most significant effect being observed after 24 h of exposure (p = 0.010). Interestingly in WT the constant exposure and pulse treatments resulted in similar increases in total number of shoots, while in lines 4 and 112 opposite effects were observed between pulse exposure and constant exposure with the trend matching after 72h exposure, suggesting a differential role between a short pulse vs prolonged exposure, and therefore a potential significant effect of an inductive vs constitutive signal. While there were differences in all lines in the morphogenetic outcomes in response to tryptophan treatment, little difference in overall growth (measured as fresh weight) was observed in response to pulse treatments, with only the 24 h pulse treatment with tryptophan leading to a significant effect, a decrease in growth (Fig 5a, 5c and 5e). This suggests tryptophan is more important in determining the growth outcome and is not just feeding into increased primary growth via an increase in the metabolic pool available for protein synthesis.

**Table 1:** Linear regression statistics for growth data (number of shoots produced per explants, percent of explants producing microshoots, number of roots per explants and percent of explants producing roots) in wild-type St. John’s wort after 24, 48 and 72 h exposure. IAA, 10 μM indole-3-acetic acid; TRP, 10 μM tryptophan; MSO, control.

Treatment	r^2^	slope	p-value
*Number of shoots*			
MSO	0.4686	-0.01465	0.52
IAA	0.9236	-0.01204	0.1783
TRP	0.7961	0.02637	0.2983
*Percent of explants with microshoots*			
MSO	0.3899	-0.3906	0.5707
IAA	0.8154	-0.4232	0.2827
TRP	0.9567	-1.237	0.1334
*Number of roots*			
MSO	0.002037	0.0001395	0.9713
IAA	0.9725	0.1003	0.1061
TRP	0.4286	-0.001953	0.5456
*Percent of explants with roots*			
MSO	0	0	>0.999
IAA	0.8463	0.7813	0.2565
TRP	0.25	-0.1302	0.6667

Note: values over 0.9 are shaded

**Fig 6:**
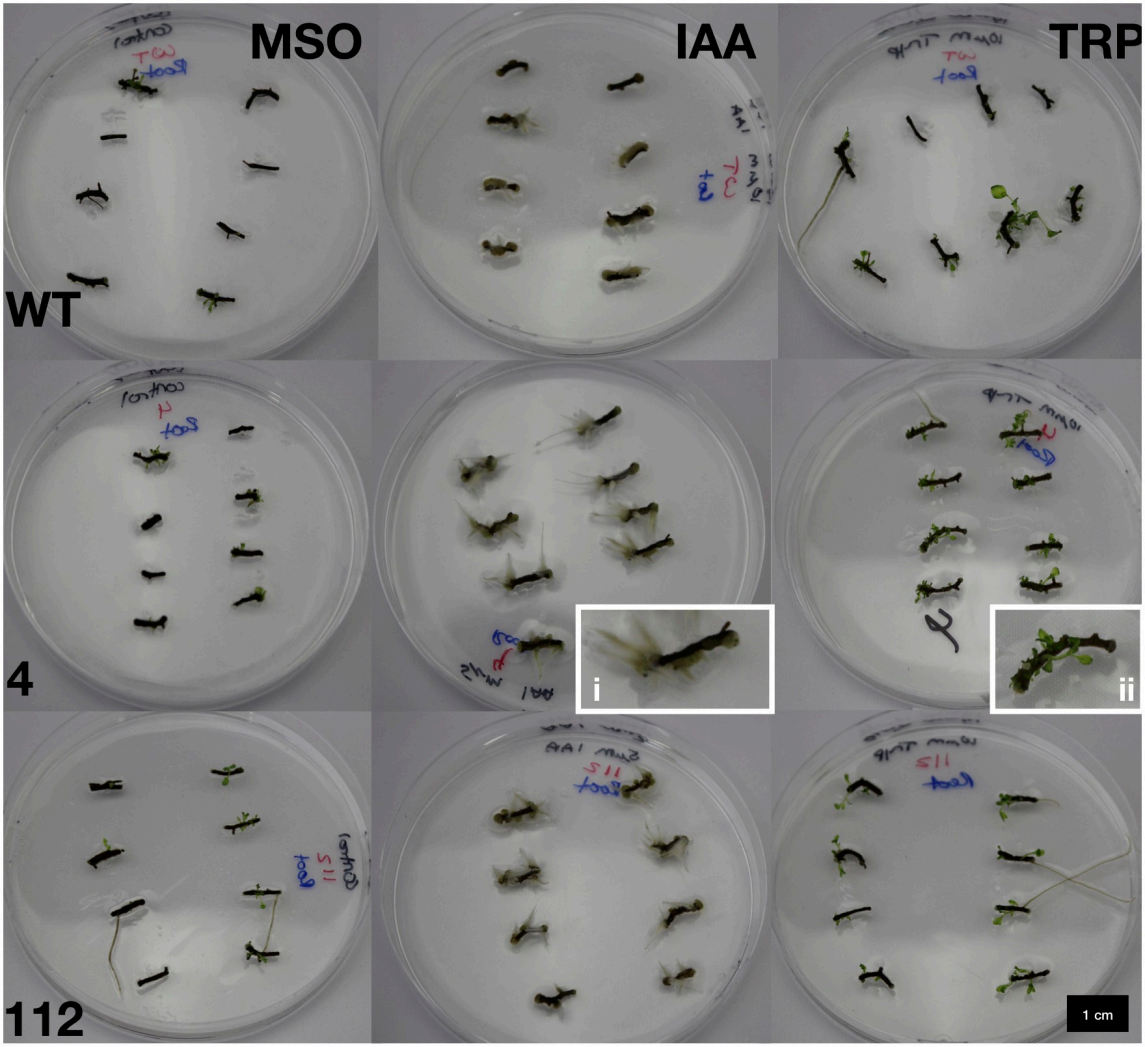
Representative growth of three lines—Wildtype (WT; top row), 4 (middle row) and 112 (bottom row)—Of St. John’s wort cultures under control conditions (left column; MSO); after exposure to auxin (centre column; indole-3-acetic acid, IAA), or tryptophan (right column; TRP).

Short inductive exposures to tryptophan did not have a significant effect on *de novo* root production but a significant increase in root organogenesis was observed in cultures of line 4 with constant exposure to tryptophan (Fig 4g and 4h, p = 0.043). Notably roots resulting from tryptophan treatment were distinct in appearance from those resulting from auxin treatment, but closely resembled those which were produced on control medium. Roots resulting from tryptophan treatment were generally longer, darker coloured roots with fewer root hairs as compared with IAA-induced roots (Fig 6). Roots resulting from tryptophan or control medium originated from the bottom side of the explant (media exposed), and generally grew into the medium, while those resulting from IAA treatment generally grew from the top of explants.

### Effects of auxin treatment on indoleamine metabolism

**Fig 7:**
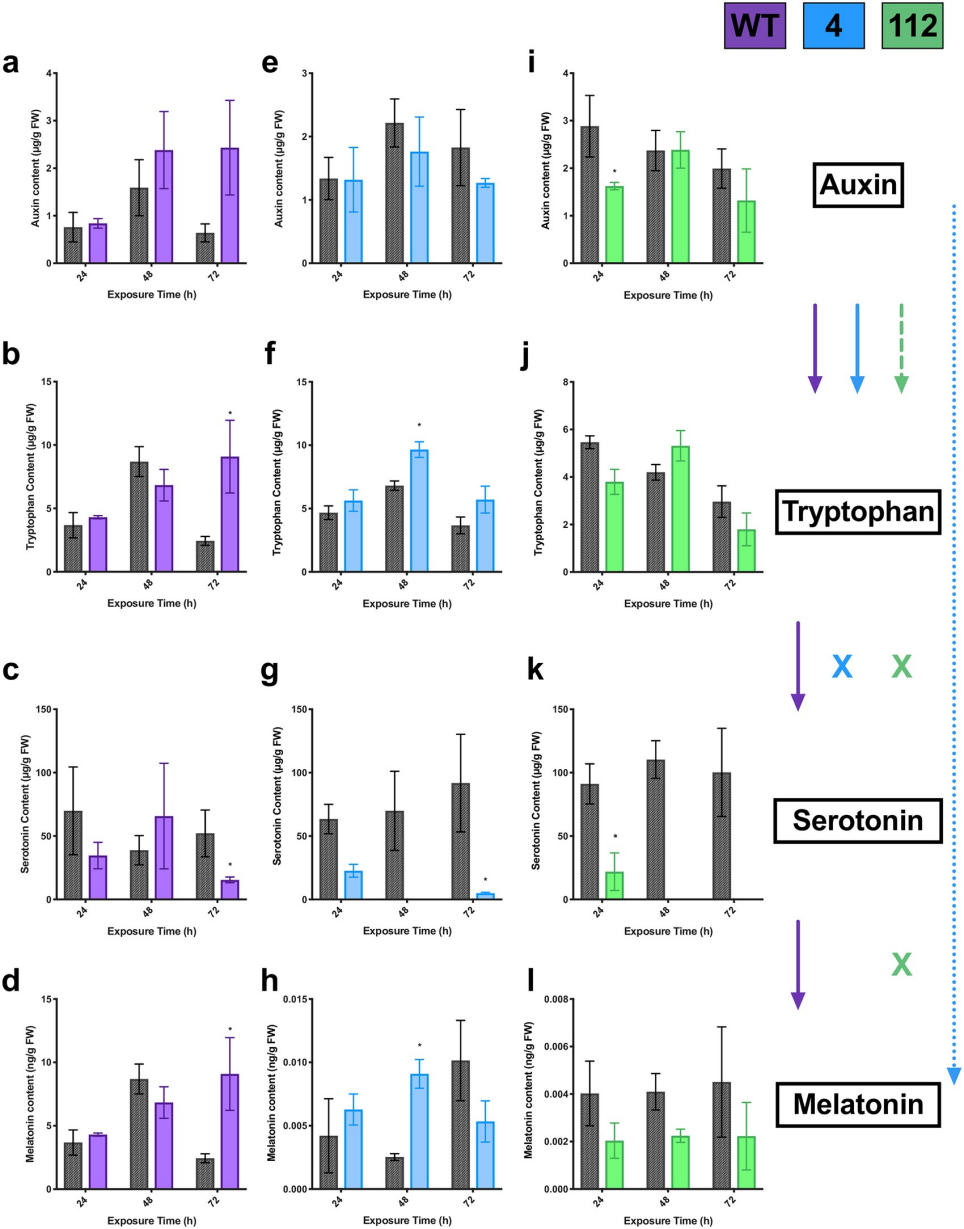
Effects of auxin (indole-3-acetic acid) treatment (10 μM) on endogenous tryptophan (b, f, j), auxin (s, e, i), serotonin (c, g, k), and melatonin (d, h, l) levels in three lines of St. John’s wort roots wildtype (WT; purple); 4 (blue) and 112 (green). * indicates treatment is significantly different than control (MSO; grey) at a given exposure time by t-test, with α = 0.05 (n = 5). Solid lines indicate tryptophan conversion to the metabolite, hashed lines are a putative conversion pathway, and ‘x’ indicates that the conversion is interrupted.

Two general themes were observed in the metabolite levels across lines in response to IAA treatment: (a) Serotonin content was universally reduced in all lines at all time points, though the effect was most significant after longer exposure periods and in lines 4 and 112 (Fig 7c, 7g and 7k); and (b) Tryptophan levels were increased in response to IAA treatment, though this was significant only in WT and line 4, at 72 and 48 h, respectively (Fig 7b, 7f and 7j). Auxin and melatonin content, interestingly, showed differential effects across the lines. In WT SJW cultures, treatment with bolus doses of IAA significantly increased tryptophan (Fig 7b), and melatonin (Fig 7d) and significantly decreased serotonin content (Fig 7c). In contrast in line 112, treatment with IAA did not significantly increase any of the metabolites measured (Fig 7i–7l). Serotonin levels were significantly reduced, to the point of being below limits of detection after 48 h (Fig 7k), while melatonin levels were also reduced (Fig 7l), though not significantly (p_48h_ = 0.057; p_24,72h_ = 0.171). Similarly, in line 4, treatment with IAA lead to a significant decrease in serotonin levels (Fig 7g), and significant increases in tryptophan (Fig 7f) and melatonin (Fig 7h) were observed. These results suggest an interaction between the auxin and indoleamine pathways.

### Effects of auxin treatment on growth and morphogenesis

**Fig 8:**
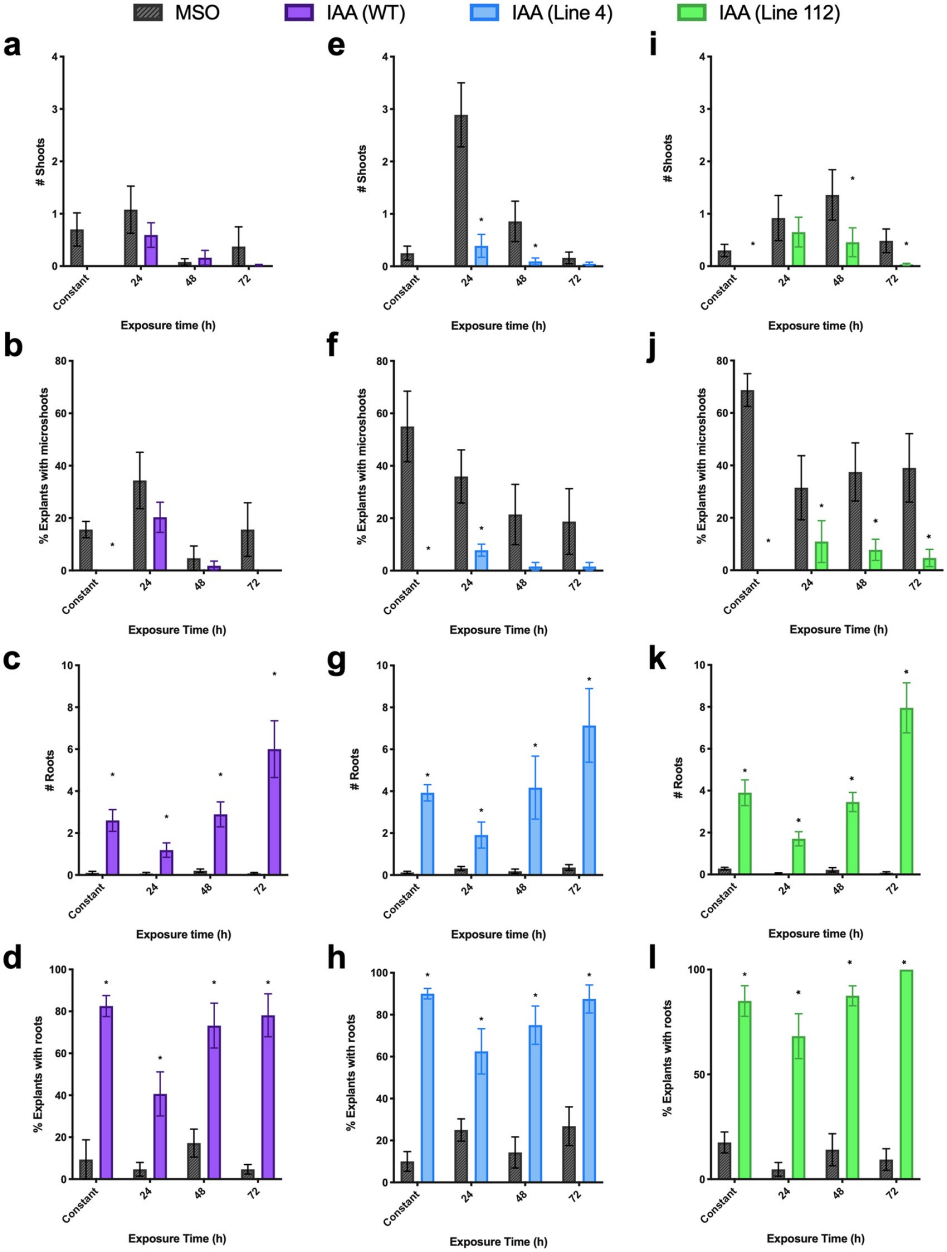
Effects of auxin (indole-3-acetic acid) treatment (10 μM) on morphogenesis as measured by mean number of shoots produced per explant (a, e, i), mean percent of explants with the presence of microshoots, defined as shoots under 1 mm in length (b, f, j), mean number of roots produced per explant (c, g, k) and mean percent of explants producing roots (d, h, l) in three lines of St. John’s wort roots: Wildtype (purple; a-d), 4 (blue; e-h) and 112 (green; i-l). * indicates treatment is significantly different than control (MSO; grey) at a given exposure time by t-test, with α = 0.05 (n = 5).

IAA decreased *de novo* shoot organogenesis in all three lines, under both constant exposure and with pulse treatment (Fig 8a, 8b, 8e, 8f, 8i and 8j). Unlike the trend in tryptophan treatment where number of shoots increased slightly after 72 h of exposure in line 4, IAA had a linear and increasingly inhibitory effect on shoot organogenesis in all three lines increasing over time (Tables 1–3).

**Table 2:** Linear regression r^2^ values for growth data (number of shoots produced per explants, percent of explants producing microshoots, number of roots per explants and percent of explants producing roots) in St. John’s wort Line 4 after 24, 48 and 72 h exposure. IAA, 10 μM indole-3-acetic acid; TRP, 10 μM tryptophan; MSO, control.

Treatment	r^2^	slope	p-value
*Number of shoots*			
MSO	0.926	-0.05687	0.1754
IAA	0.850	-0.007161	0.2531
TRP	0.1028	-0.003953	0.7922
*Percent of explants with microshoots*			
MSO	0.8636	-0.3581	0.2408
IAA	0.75	-0.1302	0.3333
TRP	0.8167	0.3627	0.2516
*Number of roots*			
MSO	0.05769	0.00009301	0.8456
IAA	0.9941	0.1090	0.0491
TRP	0.01976	-0.0002325	0.9102
*Percent of explants with roots*			
MSO	0.01744	0.03720	0.9157
IAA	1	0.5208	-
TRP	0.75	0.1721	0.3333

Note: values over 0.9 are shaded

**Table 3:** Linear regression r^2^ values for growth data (number of shoots produced per explants, percent of explants producing microshoots, number of roots per explants and percent of explants producing roots) in St. John’s wort Line 112 after 24, 48 and 72 h exposure. IAA, 10 μM indole-3-acetic acid; TRP, 10 μM tryptophan; MSO, control.

Treatment	r^2^	slope	p-value
*Number of shoots*			
MSO	0.2475	-0.009068	0.6685
IAA	0.9556	-0.01287	0.1352
TRP	0.9891	-0.03809	0.0666
*Percent of explants with microshoots*			
MSO	0.8966	0.1581	0.2084
IAA	1	-0.1302	-
TRP	0.3067	-0.4464	0.6264
*Number of roots*			
MSO	0.02913	0.0006510	0.8908
IAA	0.9397	0.1302	0.1579
TRP	0.1579	-0.000651	0.7399
*Percent of explants with roots*			
MSO	0.25	0.09766	0.6667
IAA	0.9851	0.6619	0.0779
TRP	0	0	>0.999

Note: values over 0.9 are shaded

In all cases, IAA increased root production significantly under both constant and pulse exposure, with 24 h pulse exposure being sufficient to significantly increase root production in all three lines (Fig 8c, 8d, 8g, 8h, 8k and 8l) with root production increasing in a dose dependent and linear manner, which was strongest in line 112 (Table 3), followed by line 4 (Table 2) then WT (Table 1). IAA treatment resulted in shorter and lighter coloured roots (Fig 6), with some turning pink or red (possibly suggesting stress). Additionally, IAA induced significantly more root hairs giving the appearance of fuzzy roots, which tended to grow from the face up side of the root explant. This increase in root growth is reflected in the significant increase in fresh weight in all lines (Fig 5b, 5d and 5f). IAA treatment had no significantly different effect on rooting between the three lines. *De novo* root organogenesis was also associated with callus production in IAA treated cultures, with the root explant in many cases being completely covered in callus, while tryptophan exposure or control plants had little to no callus formation (Fig 6).

## Discussion

The objective of this study was to understand the mechanisms and control of plant morphogenesis by tryptophan, auxin, melatonin and serotonin in three lines of SJW. SJW was selected for this study as the three lines examined have previously been found to respond differentially to exogenous treatment (constant exposure) with tryptophan [26].

Tryptophan is a well-established precursor of two important and now established classes of plant growth regulators: (a) the classical phytohormone auxin which is produced from tryptophan via both tryptamine dependent and independent pathways [49] and (b) the indoleamines melatonin and serotonin which are produced, in plants, via conversion of tryptophan first to tryptamine [2]. Auxin is characterized by its role in maintaining apical dominance and gravitropism as well as many other important functions, and in tissue culture auxin is characterized by its induction of root growth [56,57]. Melatonin and serotonin in contrast appear to have subtler but still expansive effects, with their strongest effects being in promoting vigour and survival upon diverse biotic and abiotic challenges. Additionally, the indoleamines also have effects on both vegetative and reproductive growth, and have been proposed to exist in balance with one another to promote root and shoot growth in plant tissue culture [2,21,22,27,28,58–60]. Proposed mechanisms of indoleamine action are diverse and are attributed to their antioxidant potential and interactions with other plant growth regulators, most notably auxin [21,23,27,31,32,34]. In this study melatonin, serotonin, auxin and tryptophan levels were quantified at the end of the growth period and after collection of all growth parameters, making them an indicator of analyte levels at the end point of the experiment and not representative of the induction signal (although this would be an interesting field for further investigation).

In general, tryptophan had a positive effect on *de novo* shoot organogenesis while IAA promoted *de novo* root organogenesis. Tryptophan was found to have differential effects on metabolite levels across the three different lines, with no increase in the tryptophan pool being observed in WT and little change seen in the downstream metabolites. The moderated effect in WT SJW, suggests that supplementary tryptophan (a) may be rapidly converted to auxin or indoleamine which are utilized or further transformation, (b) feeds into increased protein synthesis, or (c) is converted to other secondary metabolite pathways. Stimulation of increased protein synthesis by increased tryptophan availability is suggested by both the marginal increase in fresh weight and the fact WT plants generally showed increased primary growth, but more limited effects on *de novo* organogenesis. An increase in primary growth is well established as being linked with increase protein and primary metabolite biosynthesis [50,61]. Interestingly across both tryptophan and auxin treatments, serotonin levels were found to be almost universally decreased by supplementation, with some exceptions, while melatonin levels varied significantly with treatment. These results were unexpected as T-5-H is a very active enzyme, with apparently relatively few controls [42]. Our previous study in SJW also found that tryptamine, and to a lesser extend tryptophan, was rapidly converted to serotonin in response to constant exposure [27]. The decreases in serotonin levels in this study suggest that serotonin is expressed transiently in response to tryptophan and may be an inductive signal as has been hypothesized in the case of indoleamine induction in response to beetle feeding in American elm (*Ulmus americana* L.) [62].

Line 4 showed both a unique growth profile as well as a unique metabolic pattern. In contrast to WT and line 112, line 4 showed greater baseline levels of shoot and root production in the control conditions (Fig 4), and generally showed inhibition of growth upon treatment. This may indicate a greater baseline regeneration potential which is related to indoleamine levels. Indoleamines are well characterized as having dose dependent effects [63], therefore treatment with the indoleamines have an inhibitory rather than a promoting effect in this line due to modified sensitivity or regulation of this pathway. Metabolically, in response to tryptophan treatment a significant increase in melatonin is observed with an associated decrease in serotonin levels in line 4 only. This is an expected result if carbon flows through this pathway, but it is interesting that this result is only present in line 4. A concomitant significant decrease in auxin levels also suggests that there may have been a reallocation of carbon in this line. SNAT and ASMT are the two enzymes in the main biosynthetic pathway which convert serotonin to melatonin. Under normal conditions these enzymes are highly regulated [64], and this prevents depletion of the serotonin pool, however, it is plausible that this line may have modified regulation of one or both of these enzymes leading to rapid conversion to NAS (not measured in this study) or melatonin. Our previous report which examined indoleamine levels in response to feeding of each intermediate found that conversion through the pathway from tryptophan was disrupted after the conversion of trypamine to serotonin with significantly decreased serotonin levels in line 4, while in contrast line 112 showed a disruption only in the final steps of conversion of serotonin to melatonin [27]. This suggests that serotonin may be an important point in the signalling cascade or growth processes with upstream tryptophan signals being disrupted in the two lines. It is important to note that melatonin biosynthesis is clearly disrupted (1000 x reduction; Fig 3d, 3h and 3l) in these lines which were derived from haploid culture and EMU mutagenesis as previously described [27,51,52]. Though the actual sites of targets/sources of these modifications are yet to be characterized in these lines, it is possible that the mutations may be in a regulatory mechanism vs in in the biosynthetic pathway itself.

Auxin (IAA) had a markedly different effect on both metabolite levels and morphogenesis than we hypothesized and indicates a previously hypothesized and important cross-talk exists between the indoleamines and auxin pathways, though previous investigations have solely focused on the effect of indoleamines on auxin transport/action vs applied auxin mediating indoleamine activity [32,65–68]. In all cases IAA treatment led to a significant increase in root morphogenesis, a classical and anticipated auxin response. Biochemically, serotonin levels were significantly reduced in all three lines, and tryptophan levels were generally increased, though the latter was only significant in WT and line 4. In both WT and line 4 this was also associated with an accumulation of melatonin, with no significant changes in IAA. This suggests some of the carbon may be being re-allocated towards indoleamine biosynthesis in response to high levels of IAA, and could possibly be explained by feedbank inhibition of auxin biosynthesis. The increase in melatonin content with a concomitant decrease in serotonin is more difficult to explain. This effect may be the result of increased availability of tryptophan as auxin biosynthesis is slowed due to feedback inhibition or it may be due to redirection of carbon towards an alternate biosynthetic pathway for melatonin which does not use serotonin as a precursor [46]. The likelihood of rapid conversion of serotonin to melatonin through the primary biosynthetic pathway is unlikely as ASMT in particular has been proposed as a rate limiting steps in the pathway [21]. A further possible explanation which has been proposed, is that IAA itself may be converted to melatonin, though there has been no evidence of this biosynthetic transformation since its proposal [69]. The modification in indoleamine levels in response to auxin treatment suggests not only that melatonin and serotonin are able to exert their effects on morphogenesis through modification of auxin signaling pathways, but that auxin also functions to modify serotonin and melatonin. This further elucidates a complex relationship between these already closely linked plant growth regulators. Though melatonin levels were not found to increase in line 112 in response to IAA supplementation, serotonin levels were again found to be reduced, this is in agreement with our previous findings that the indoleamine pathway is disrupted between serotonin and melatonin [27], and suggests that there is reduced carbon flow through the pathway. Interestingly, no difference in morphogenetic or growth outcomes were observed between the pulse and the constant treatment with auxin as was observed in tryptophan supplementation.

The results from both IAA and tryptophan exposure suggest that (1) plants respond to short pulses of tryptophan differentially, (2) tryptophan functions as an inductive signal in plants, and (3) inductive signals from tryptophan or plant growth regulators, such as IAA, can trigger the morphogenetic cascade with even very brief exposure times. The differences in morphogenetic outcomes of constant vs pulse treatment of explants between tryptophan and auxin may have several explanations. It is possible that auxin, which has specific receptors and signaling cascades, has a more consistent effect on growth than tryptophan which may feed into different pathways. Therefore, tryptophan may have many more possible outcomes as an inductive signal or may have a weaker inductive signal, as shown by the strong and consistent promotion of shoot organogenesis in response to constant exposure as compared to the variable effects it has between the lines and across inductive periods (e.g. 24 vs 48 vs 72 h).

## Conclusions

Together these results suggest plants differentially switch flow of tryptophan from one downstream pathway to another. This is a not uncommon occurrence in plants as supply of common precursors into many diverse pathways must be strictly controlled in many instances. Thus, our original hypothesis, that the observed growth effects of tryptophan are solely due to its status as a precursor for other plant growth regulating pathways, needs revision. We propose that tryptophan serves as a point of cross talk between the melatonin and auxin biosynthetic pathways, playing a role in their complex interactions which fine tune the symphony of plant morphogenesis. Furthermore, we show that a feature of tryptophan-mediated morphogenesis is a dual function as both a common precursor and as a plant growth regulator (or inductive signal). Additionally, we highlight a potential new minor mechanism for auxin action, the mediation of indoleamine metabolism, which requires further investigation. Sequencing, characterization and identification of the mutations in the lines employed in this study will allow for a better understanding of the interaction between auxin and the indoleamines, and may also serve to elucidate the role of tryptophan as both an inductive signal and a precursor and the importance this function may serve in mediating transient indoleamine growth or stress signals.

## Supporting information

S1 Table Summary of p-values resulting from t-test to determine if endogenous tryptophan, melatonin, serotonin or indole-3-acetic acid (IAA) content in treated (tryptophan or auxin) St. John’s wort roots is significantly different from control (MSO), n = 5, α = 0.05. Significant results are in bold.
(DOCX)

S2 Table Summary of p-values resulting from t-test to determine if growth effects in treated (tryptophan or auxin) St. John’s wort roots is significantly different from control (MSO), n = 5, α = 0.05. Significant results are in bold.
(DOCX)
