# Supplementary material for: Auxin driven indoleamine biosynthesis and the role of tryptophan as an inductive signal in Hypericum perforatum (L.)
Source: PLoS One. 2019 Oct 17;14(10):e0223878. doi: 10.1371/journal.pone.0223878 (PMC6797091; doi:10.1371/journal.pone.0223878)
Supplement: S1 Table — Significant results are in bold. (DOCX) [file pone.0223878.s001.docx]

**S1 Table. Summary of p-values resulting from t-test to determine if endogenous concentrations of tryptophan, melatonin, serotonin or indole-3-acetic acid (IAA) content in treated (tryptophan or auxin) St. John’s wort roots is significantly different from control (MSO),** n=5, α = 0.05. Significant results are in bold.

|  | | WT | 4 | 112 |
| --- | --- | --- | --- | --- |
| *Tryptophan Content* | |  |  |  |
| Tryptophan | 24h | 0.343 | **0.029** | **0.014** |
|  | 48h | 0.243 | 0.114 | **0.014** |
|  | 72h | 0.114 | 0.171 | **0.029** |
| IAA | 24h | 0.343 | 0.243 | 0.057 |
|  | 48h | 0.314 | **0.029** | 0.100 |
|  | 72h | **0.014** | 0.100 | 0.243 |
| *IAA Content* | |  |  |  |
| Tryptophan | 24h | 0.343 | **0.029** | 0.443 |
|  | 48h | 0.443 | 0.200 | 0.057 |
|  | 72h | 0.314 | 0.100 | 0.343 |
| IAA | 24h | 0.343 | 0.429 | **0.014** |
|  | 48h | 0.200 | 0.429 | 0.443 |
|  | 72h | 0.100 | 0.500 | 0.171 |
| *Melatonin Content* | |  |  |  |
| Tryptophan | 24h | 0.243 | 0.314 | 0.443 |
|  | 48h | 0.243 | **0.029** | 0.343 |
|  | 72h | 0.114 | **0.029** | 0.100 |
| IAA | 24h | 0.343 | 0.314 | 0.171 |
|  | 48h | 0.314 | **0.029** | 0.057 |
|  | 72h | **0.014** | 0.100 | 0.171 |
| *Serotonin Content* | | WT | 4 | 112 |
| Tryptophan | 24h | 0.500 | 0.100 | 0.100 |
|  | 48h | 0.057 | 0.314 | 0.343 |
|  | 72h | 0.500 | 0.500 | 0.500 |
| IAA | 24h | 0.443 | 0.1714 | **0.029** |
|  | 48h | 0.400 | **nd** | **nd** |
|  | 72h | **0.029** | **nd** | **nd** |
