# Supplementary material for: Auxin driven indoleamine biosynthesis and the role of tryptophan as an inductive signal in Hypericum perforatum (L.)
Source: PLoS One. 2019 Oct 17;14(10):e0223878. doi: 10.1371/journal.pone.0223878 (PMC6797091; doi:10.1371/journal.pone.0223878)
Supplement: S2 Table — Significant results are in bold. (DOCX) [file pone.0223878.s002.docx]

**S2 Table. Summary of p-values resulting from t-test to determine if growth effects in treated (tryptophan or IAA) St. John’s wort roots is significantly different from control (MSO),** n=5, α = 0.05. Significant results are in bold.

|  | | | | WT | | 4 | | 112 |
| --- | --- | --- | --- | --- | --- | --- | --- | --- |
| *Mean number of shoots/explant* | | | |  | |  | |  |
| Tryptophan | | Constant | | **0.048** | | **0.040** | | **0.004** |
|  |  | 24h | | 0.854 | | **0.010** | | 0.123 |
|  |  | 48h | | 0.106 | | 0.061 | | 0.432 |
|  |  | 72h | | 0.569 | | 0.217 | | 0.438 |
| IAA | | Constant | | **0.024** | | **0.037** | | **0.019** |
|  |  | 24h | | 0.370 | | **0.006** | | 0.268 |
|  |  | 48h | | 0.446 | | **0.027** | | **0.048** |
|  |  | 72h | | 0.361 | | 0.277 | | **0.016** |
| *Mean percent of explants with microshoots* | | | | | |  | |  |
| Tryptophan | | Constant | | 0.294 | | 0.100 | | **0.016** |
|  |  | 24h | | **0.016** | | **0.018** | | 0.128 |
|  |  | 48h | | **0.008** | | 0.198 | | 0.067 |
|  |  | 72h | | 0.406 | | 0.099 | | 0.366 |
| IAA | | Constant | | **0.005** | | **0.002** | | **0.005** |
|  |  | 24h | | 0.229 | | 0.052 | | **0.044** |
|  |  | 48h | | 0.500 | | 0.077 | | **0.033** |
|  |  | 72h | | 0.105 | | 0.221 | | **0.036** |
| *Mean number of roots/explant* | | | |  | |  | |  |
| Tryptophan | | Constant | | 0.476 | | **0.043** | | 0.476 |
|  |  | 24h | | 0.143 | | 0.260 | | 0.112 |
|  |  | 48h | | 0.226 | | 0.465 | | 0.383 |
|  |  | 72h | | 0.314 | | 0.500 | | 0.392 |
| IAA | | Constant | | **0.013** | | **0.025** | | 0.065 |
|  |  | 24h | | **0.011** | | **0.200** | | **0.002** |
|  |  | 48h | | **0.004** | | **0.034** | | **0.002** |
|  |  | 72h | | **0.001** | | **0.005** | | **0.0005** |
| *Mean number of roots/explant* | | | |  | |  | |  |
| Tryptophan | | Constant | | 0.595 | | **0.013** | | 0.460 |
|  |  | 24h | | 0.053 | | 0.586 | | 0.223 |
|  |  | 48h | | 0.206 | | 0.707 | | 0.120 |
|  |  | 72h | | 0.103 | | 0.985 | | 0.500 |
| IAA | | Constant | | **0.008** | | **0.004** | | **0.004** |
|  |  | 24h | | **0.008** | | **0.004** | | **0.001** |
|  |  | 48h | | **0.001** | | **0.0006** | | **0.0002** |
|  |  | 72h | | **<0.0001** | | **0.0005** | | **<0.0001** |
| *Mean fresh weight* | | | WT | | 4 | | 112 | |
| Tryptophan | 24 h | | 0.389 | | **0.00040** | | 0.746 | |
|  | 48 h | | 0.298 | | 0.231 | | 0.853 | |
|  | 72 h | | 0.719 | | 0.244 | | 0.746 | |
| IAA | 24 h | | 0.232 | | 0.416 | | **0.040** | |
|  | 48 h | | 0.232 | | 0.232 | | **0.040** | |
|  | 72 h | | **0.0011** | | **0.0043** | | **0.0000052** | |
